# Early Changes in Optic Nerve Sheath Diameter Associated with Endotracheal Tube Versus Laryngeal Mask Airway in Children Undergoing Elective Surgery Under General Anesthesia: A Prospective Observational Study

**DOI:** 10.3390/jcm15218345

**Published:** 2026-10-27

**Authors:** Dilara Göçmen, Feyza Özaltun, Yasemin Masatlıoğlu, Canan Topcu, Tümay Umuroğlu

**Affiliations:** Department of Anesthesiology and Reanimation, Marmara University Pendik Training and Research Hospital, Marmara University School of Medicine, Istanbul 34899, Türkiye

**Keywords:** optic nerve, ultrasonography, monitoring, physiologic, intubation, intratracheal, laryngeal masks, child, anesthesia, general, intracranial pressure

## Abstract

**Purpose:** Ultrasonographic optic nerve sheath diameter (ONSD) has been investigated as a noninvasive sonographic marker associated with intracranial pressure. This study aimed to compare the early change in ONSD after airway placement between children managed with an endotracheal tube (ETT) and those managed with a laryngeal mask airway (LMA). **Methods:** This single-center prospective, non-randomized observational study included 160 children stratified into age groups of 1–4 and 5–11 years, with 40 patients in each airway subgroup. Bilateral mean ONSD was measured before airway placement (T0), immediately after airway placement and initiation of mechanical ventilation (T1), and after airway removal but before full awakening (T2). The primary outcome was the between-device difference in the change in ONSD from T0 to T1. Repeated measurements were analyzed using a linear mixed-effects model, with Holm adjustment for multiple comparisons. **Results:** The increase in bilateral mean ONSD from T0 to T1 was 0.269 mm greater in the ETT group than in the LMA group (95% confidence interval [CI], 0.131–0.408; Holm-adjusted *p* < 0.001). The age-specific ETT–LMA difference in change was 0.293 mm in children aged 1–4 years (95% CI, 0.097–0.488; Holm-adjusted *p* = 0.0068) and 0.246 mm in those aged 5–11 years (95% CI, 0.050–0.442; Holm-adjusted *p* = 0.0137). The age group × airway device × time interaction was not significant (Holm-adjusted *p* = 0.743). In exploratory analyses, peak inspiratory and plateau pressures were higher in the ETT group in both age strata. **Conclusions:** In the early period after airway placement, ETT use was associated with a greater increase in bilateral mean ONSD than LMA use. Although the association was directionally consistent in both age strata, there was no evidence that the magnitude of the effect differed by age group. Because ONSD is not a direct measurement of intracranial pressure, these findings should not be interpreted as evidence of clinical intracranial hypertension or causality. The airway device was not randomized but was determined by surgical and clinical factors and the attending anesthesiologist’s judgment, and the clinical significance of the 0.269 mm difference is uncertain.

## 1. Introduction

The endotracheal tube (ETT) and laryngeal mask airway (LMA) are widely used airway devices during pediatric general anesthesia. Direct laryngoscopy and tracheal stimulation may transiently affect cerebral venous drainage and intracranial dynamics by increasing the sympathoadrenal response, intrathoracic pressure, and central venous pressure [1,2]. Because LMA placement does not require laryngoscopy and does not directly stimulate the tracheal mucosa, it is generally associated with a less pronounced physiological response [3,4].

The subarachnoid space surrounding the optic nerve is continuous with the intracranial subarachnoid space. Optic nerve sheath diameter (ONSD), measured by bedside ultrasonography, is a noninvasive measure used in the assessment of intracranial hypertension [5,6]. An association between ultrasonographic ONSD and invasively measured intracranial pressure has been reported in adults [7]. In pediatric patients, both ONSD and its temporal change have also been reported as potential indicators of intracranial hypertension [8,9,10]. However, pediatric ONSD is influenced by age, transducer placement, imaging plane, and operator experience, and no single threshold is applicable to all age groups [11]. Consequently, temporal change assessed within the same patient using a standardized measurement technique may be more informative than a single absolute value [12]. However, even with a standardized technique, serial ONSD measurements are not immune to measurement error. Whether a small, transient between-device difference in perioperative ONSD is physiologically or clinically meaningful is uncertain, because ONSD is an indirect marker whose short-term perioperative changes have not been linked to clinical outcomes.

An increase in ONSD after laryngoscopy and tracheal intubation has been reported in a limited number of adult studies [13,14,15]. In adults, i-gel use has been associated with lower hemodynamic and intraocular pressure responses than endotracheal intubation [16]. However, short-term changes in ONSD after ETT and LMA placement have not been directly compared across different pediatric age strata. Concurrent ventilatory and physiological monitoring data may help characterize the clinical context of the observed change in ONSD.

The primary objective of this study was to determine the difference between the ETT and LMA groups in the change in bilateral mean ONSD from T0 to T1 in children aged 1–11 years. Secondary objectives were to assess whether this association differed between the 1–4- and 5–11-year age strata, to evaluate changes from baseline to T2 and from T1 to T2, and to examine consistency between right- and left-eye measurements. Intraoperative physiological and ventilatory variables were analyzed as exploratory outcomes. We hypothesized that ETT use would be associated with a greater early increase in ONSD than LMA use.

## 2. Materials and Methods

### 2.1. Study Design and Ethics

This single-center prospective observational study was conducted in the operating rooms of the Asaf Ataseven Additional Service Building of Marmara University Pendik Training and Research Hospital between 1 June 2025 and 1 June 2026 and was reported in accordance with the STROBE recommendations. The study was registered at ClinicalTrials.gov (NCT06994481; registration date: 20 May 2025).

The study was approved by the Marmara University School of Medicine Ethics Committee for Non-Drug and Non-Medical Device Research (decision no. 09.2025.25-0158; approval date: 21 February 2025) and was conducted in accordance with the Declaration of Helsinki. Written informed consent was obtained from the children’s parents or legal guardians.

### 2.2. Participants

Children aged 1–11 years with an American Society of Anesthesiologists physical status of I or II who were scheduled for elective surgery under general anesthesia were eligible. Exclusion criteria were a tracheostomy; a history of intracranial tumor or shunt surgery; a neurological condition that could affect intracranial pressure; an ocular or craniofacial abnormality that precluded reliable ocular ultrasonography; and absence of consent. Patients were enrolled consecutively, and four-year-old children were assigned to the 1–4-year stratum. In accordance with the registered eligibility criteria, patients who required airway-device resizing or a second insertion or intubation attempt—including conversion from an LMA to an ETT—were prespecified exclusions and were removed before analysis rather than reclassified.

Patients were stratified into age groups of 1–4 and 5–11 years; 40 patients were analyzed in each LMA and ETT subgroup, yielding a total sample of 160. The airway device was not randomized and was selected according to surgical characteristics, the patient’s clinical condition, and the routine clinical judgment of the attending anesthesiologist. Tracheal intubation was performed using an appropriately sized Macintosh blade and a cuffed Portex endotracheal tube selected according to body weight. An appropriately sized i-gel was used in the LMA group. All patients received a neuromuscular blocking agent. The i-gel supraglottic airway (SGA) size was selected according to the manufacturer’s weight-based recommendations, and the endotracheal tube size according to our institutional age-based formula (age/4 + 3.5 for cuffed tubes), adjusted after a leak test. For consistency with the protocol the abbreviation “LMA” is retained, although the device used was the i-gel, a second-generation SGA.

### 2.3. Anesthesia and Measurements

Standard monitoring included electrocardiography, noninvasive arterial blood pressure, peripheral oxygen saturation (SpO_2_), end-tidal carbon dioxide (EtCO_2_), and bispectral index. Anesthesia was induced with propofol or thiopental and rocuronium. Anesthesia was maintained with sevoflurane and remifentanil in a mixture of 40% oxygen and 60% air; the sevoflurane concentration was adjusted to maintain a bispectral index of 40–60. Ventilator settings were adjusted by the attending anesthesiologist according to clinical requirements, with EtCO_2_ targeted at 35–45 mmHg. Heart rate, mean arterial pressure (MAP), SpO_2_, EtCO_2_, peak inspiratory pressure, positive end-expiratory pressure (PEEP), and plateau pressure were recorded initially; at 5–15, 15–25, 25–35, 35–45, and 45–55 min; and at the end of anesthesia.

### 2.4. Optic Nerve Sheath Diameter Measurements

ONSD measurements were performed by two investigators trained in the technique. One investigator performed the ultrasonographic measurement, while the other observed the procedure and recorded the obtained value. After the closed upper eyelid had been covered with a sterile transparent drape, ultrasound gel was applied and measurements were obtained using a 6–13 MHz linear transducer (MyLab™ Seven, Esaote, Genoa, Italy). An adequate image was acquired in the transverse plane and frozen. ONSD was measured 3 mm behind the posterior wall of the globe as the distance between the inner margins of the optic nerve sheath. At each time point, one measurement was obtained from each eye; the arithmetic mean of the two values was calculated as the bilateral mean ONSD. The investigator performing the measurement was not blinded to the airway device. Measurements were obtained at three time points: after induction of anesthesia and immediately before airway placement (T0); within approximately 1 min after device insertion and the start of mechanical ventilation (T1); and after device removal, while the patient was breathing spontaneously or receiving face-mask support but before full awakening (T2). The two investigators (F.Ö. and Y.M.) were trained in ONSD measurement and had each performed at least 30 examinations before study initiation. The head was kept in a neutral position with the head of the bed flat during measurement. Images were acquired but not systematically archived for offline re-analysis, and the two investigators did not measure overlapping patients; consequently, intra- and inter-observer reliability (ICC, Bland–Altman) could not be quantified.

### 2.5. Outcomes

The primary outcome was the difference between the ETT and LMA groups in the change in bilateral mean ONSD from T0 to T1. Whether the association between airway device and this early change differed by age group was assessed using a contrast corresponding to the age group × airway device × time interaction. Differences in change from T0 to T2 and from T1 to T2, together with analyses of right and left ONSD, were defined as secondary outcomes; intraoperative physiological and ventilatory variables were considered exploratory outcomes.

### 2.6. Sample Size

Before study initiation, the sample size was calculated using G*Power version 3.1.9.7 on the basis of the group × time interaction in a repeated-measures analysis of variance. The calculation assumed four groups, three repeated measurements, a correlation of 0.50 among repeated measurements, a nonsphericity correction ε of 1, a two-sided α of 0.05, and 95% power (1 − β). Assuming a small-to-moderate interaction effect of Cohen’s f = 0.15, the required sample size was 160 patients, corresponding to 40 patients per group (actual power, 95.3%). Although the sample size calculation was based on a repeated-measures ANOVA framework, a linear mixed-effects model was used for the primary analysis to model within-patient correlation directly. Ethics-committee approval was initially obtained for 200 children; before recruitment and without inspection of any outcome data, the power assumptions were revised, indicating that 160 participants (40 per cell; 95% power) were sufficient, and the ClinicalTrials.gov record and target were updated accordingly. No interim analysis of the primary endpoint was performed. The power analysis was based on the omnibus group × time interaction rather than the specific T0–T1 ETT–LMA difference-in-change contrast that served as the primary analysis; we therefore acknowledge that the study was not formally powered for this particular contrast.

### 2.7. Statistical Analysis

Continuous variables are presented as mean ± standard deviation, and categorical variables as number and percentage [n (%)]. The distribution of continuous variables was assessed using the Shapiro–Wilk test, and homogeneity of variance using Levene’s test. Categorical variables were analyzed using Pearson’s chi-square test; a Monte Carlo approach was used for the comparison of surgical types because of sparse cells. Repeated ONSD and intraoperative measurements were analyzed using linear mixed-effects models with a patient-level random intercept. Age group, airway device, categorical time, and all corresponding two- and three-way interactions were included as fixed effects. Models were estimated using restricted maximum likelihood (REML), and the distribution and homogeneity of variance of the residuals were assessed graphically. Model-based estimated marginal means and linear contrasts were calculated. A positive ETT–LMA contrast indicated a greater increase in ONSD in the ETT group over the relevant interval. Results are presented as estimated differences, 95% confidence intervals (CIs), raw *p* values, and Holm-adjusted *p* values. Holm adjustment was applied within families of analyses addressing similar scientific questions: the overall T0–T1 contrast and the test of effect modification by age constituted the confirmatory family; the T0–T1 contrasts in the two age strata constituted the age-specific family; and the age-specific T0–T2 and T1–T2 contrasts constituted the secondary ONSD family. Right- and left-eye ONSD analyses and the seven intraoperative outcomes were adjusted within their respective analysis families. A two-sided Holm-adjusted *p* < 0.05 was considered statistically significant. Missing repeated measurements were not imputed; the mixed models used all available observations. Analyses were conducted in Python 3.12.14 using pandas 2.2.3, NumPy 2.3.5, and SciPy 1.17.0. *p* values were based on two-sided Wald z tests. Because surgical type was imbalanced between groups, a post hoc sensitivity analysis restricted to urological procedures was performed. The primary mixed model was specified as ONSD ~ age group × airway device × time, including all two- and three-way interactions (reference categories: age group = 1–4 years, airway = LMA, time = T0) with a patient-level random intercept; a random-slope model was also fitted and, although it improved fit (likelihood-ratio test *p* = 0.003), the primary estimate was unchanged. Wald z-based inference was considered adequate for n = 160. To address potential confounding by surgical case-mix, the T0–T1 change difference was re-estimated in a sensitivity model adjusted for age, body weight, sex, and surgical category. Mixed-effects models and the derived contrasts were computed with Python statsmodels 0.14.5. The within-patient covariance was modeled as exchangeable through the random intercept (intraclass correlation ≈ 0.54), with homoscedastic residual variance. The model was estimated by REML and converged without singularity. Residual diagnostics indicated approximately normal residuals (Shapiro–Wilk W = 0.99) with only minor departures; a small number of standardized residuals (9 of 480) modestly exceeded ±3, no single influential observation was identified, and excluding extreme values did not change the primary estimate. Because the mixed models were estimated by REML without finite-sample degrees-of-freedom corrections, inference was based on Wald z tests; given 160 patients and 480 observations, the normal approximation was considered adequate, and t- or Satterthwaite-based corrections would be expected to have negligible impact. Missing data were limited to late intraoperative readings (e.g., end-of-anaesthesia ventilatory pressures, which were unavailable in a minority of patients); the primary ONSD measurements at T0, T1, and T2 were complete for all 160 analyzed participants.

Generative artificial intelligence (AI)-assisted technology was used during the preparation of this manuscript for English-language editing only. It was not used to generate study data, to perform or design the statistical analyses, or to produce any scientific content or conclusions. The authors reviewed and edited all AI-assisted text and take full responsibility for the final content of the manuscript. Product details of the tool are provided in the Acknowledgments.

## 3. Results

### 3.1. Participants and Baseline Characteristics

Of the 177 patients assessed for eligibility, four declined participation. Eight patients who required conversion from an LMA to an ETT, three who required repeated intubation attempts, and two in whom reliable ONSD measurements could not be obtained were excluded. Thus, 160 patients were included in the analysis.

**Figure 1:**
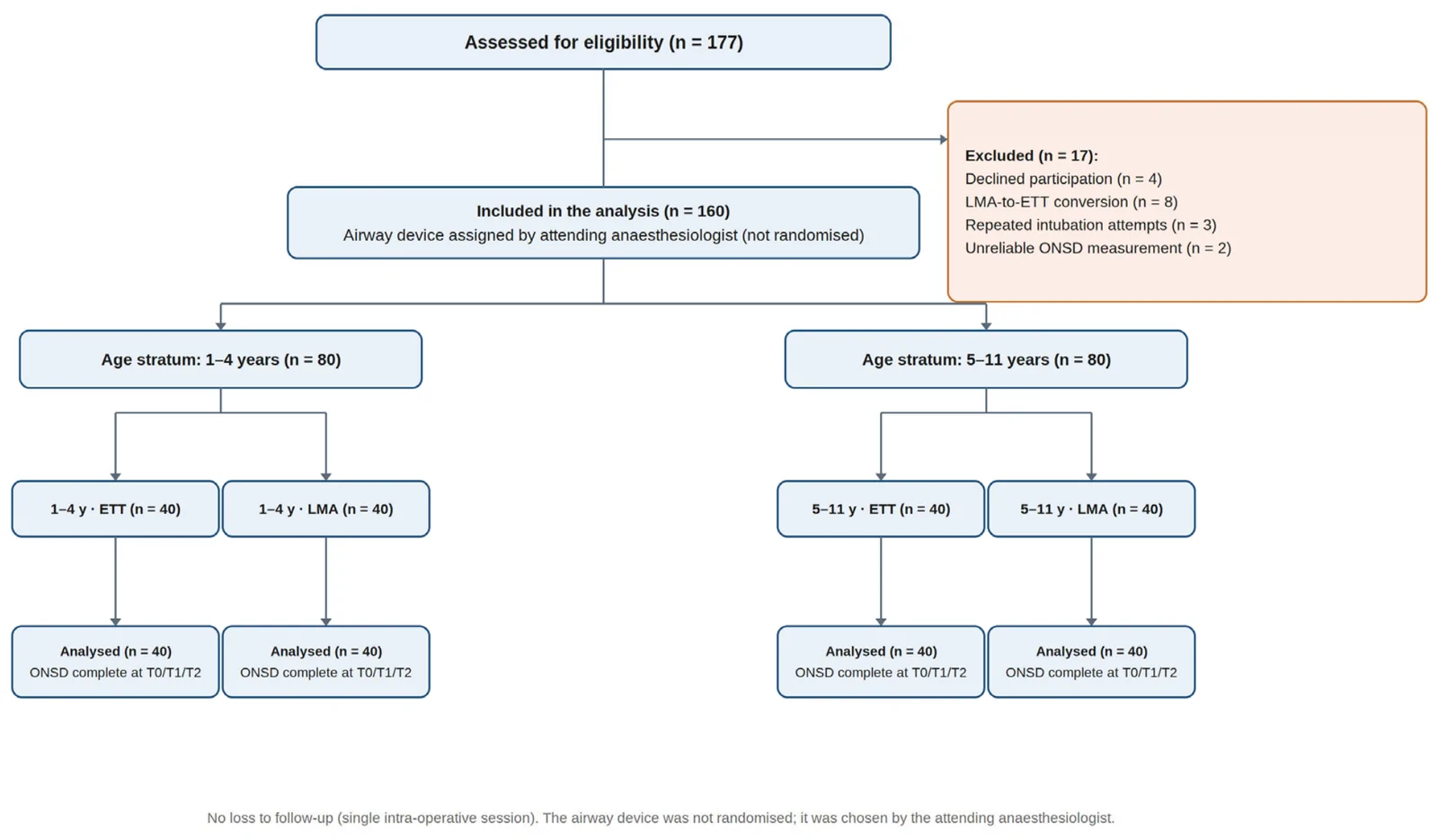
Study flow diagram. Of 177 children assessed for eligibility, 17 were excluded (4 declined participation; 8 LMA-to-ETT conversions; 3 repeated intubation attempts; 2 unreliable ONSD measurements), leaving 160 analyzed in four balanced groups. The airway device was not randomized; it was chosen by the attending anesthesiologist. ONSD was complete at T0, T1, and T2 in all analyzed patients, with no loss to follow-up (single intra-operative session).

A total of 160 patients, 40 in each of the four study groups, were analyzed; T0, T1, and T2 measurements were complete for the primary ONSD analysis. Sex distributions appeared similar across groups, whereas body weight was lower in the ETT group in both age strata. Baseline bilateral mean ONSD values were descriptively similar across groups (Table 1). Baseline imbalance described by standardized mean differences (SMD) was moderate for body weight (SMD = 0.25) and marked for surgical category: all 23 ENT cases were in the ETT groups (SMD = 0.82), whereas SMDs for age and baseline ONSD were small (0.15 and 0.14, respectively). Participant flow is summarized in Figure 1.

The distribution of surgical types differed among the groups (χ^2^ = 40.79; df = 9; Monte Carlo *p* < 0.001); ear, nose, and throat procedures occurred only in the ETT groups (Table 2).

### 3.2. Primary and Secondary ONSD Outcomes

Estimated marginal means from the linear mixed-effects model are presented in Table 3, and model-based contrasts in Table 4.

In the primary comparison, the increase in bilateral mean ONSD from T0 to T1 was 0.269 mm greater in the ETT group than in the LMA group (95% CI, 0.131–0.408; Holm-adjusted *p* < 0.001). The age-specific ETT–LMA difference in change was 0.293 mm in children aged 1–4 years (95% CI, 0.097–0.488; Holm-adjusted *p* = 0.0068) and 0.246 mm in those aged 5–11 years (95% CI, 0.050–0.442; Holm-adjusted *p* = 0.0137). The between-age-group difference in the T0–T1 ETT–LMA contrast was −0.046 mm (95% CI, −0.323 to 0.231; Holm-adjusted *p* = 0.743); therefore, there was no evidence that the early association with airway device differed by age group. In a sensitivity model adjusted for age, body weight, sex, and surgical category, the T0–T1 ETT–LMA difference was attenuated but remained significant (0.195 mm; 95% CI 0.042–0.349; *p* = 0.013). The within-group T0–T1 change was +0.314 mm (95% CI 0.207–0.422) in the ETT group and +0.045 mm (95% CI −0.046–0.136) in the LMA group.

In the post hoc sensitivity analysis restricted to urological procedures, the overall T0–T1 ETT–LMA difference in change remained in the same direction (0.221 mm; 95% CI, 0.054–0.388; nominal *p* = 0.010). In this subgroup, the device distribution was more balanced (ETT n = 47, i-gel n = 76), and the difference remained consistent in direction and significance after adjustment for age, body weight, and sex. This post hoc analysis reduces but does not eliminate residual confounding and does not establish a causal relationship.

In the secondary analyses, the ETT–LMA difference in change from T0 to T2 was 0.445 mm in children aged 1–4 years (95% CI, 0.143–0.747; Holm-adjusted *p* = 0.0157) and 0.266 mm in those aged 5–11 years (95% CI, −0.036 to 0.569; Holm-adjusted *p* = 0.253). The corresponding difference in change from T1 to T2 was 0.153 mm in children aged 1–4 years (95% CI, −0.139 to 0.444; Holm-adjusted *p* = 0.610) and 0.020 mm in those aged 5–11 years (95% CI, −0.271 to 0.311; Holm-adjusted *p* = 0.893). After adjustment, statistical significance was observed only for the T0–T2 comparison in the 1–4-year age group (Table 4).

**Figure 2:**
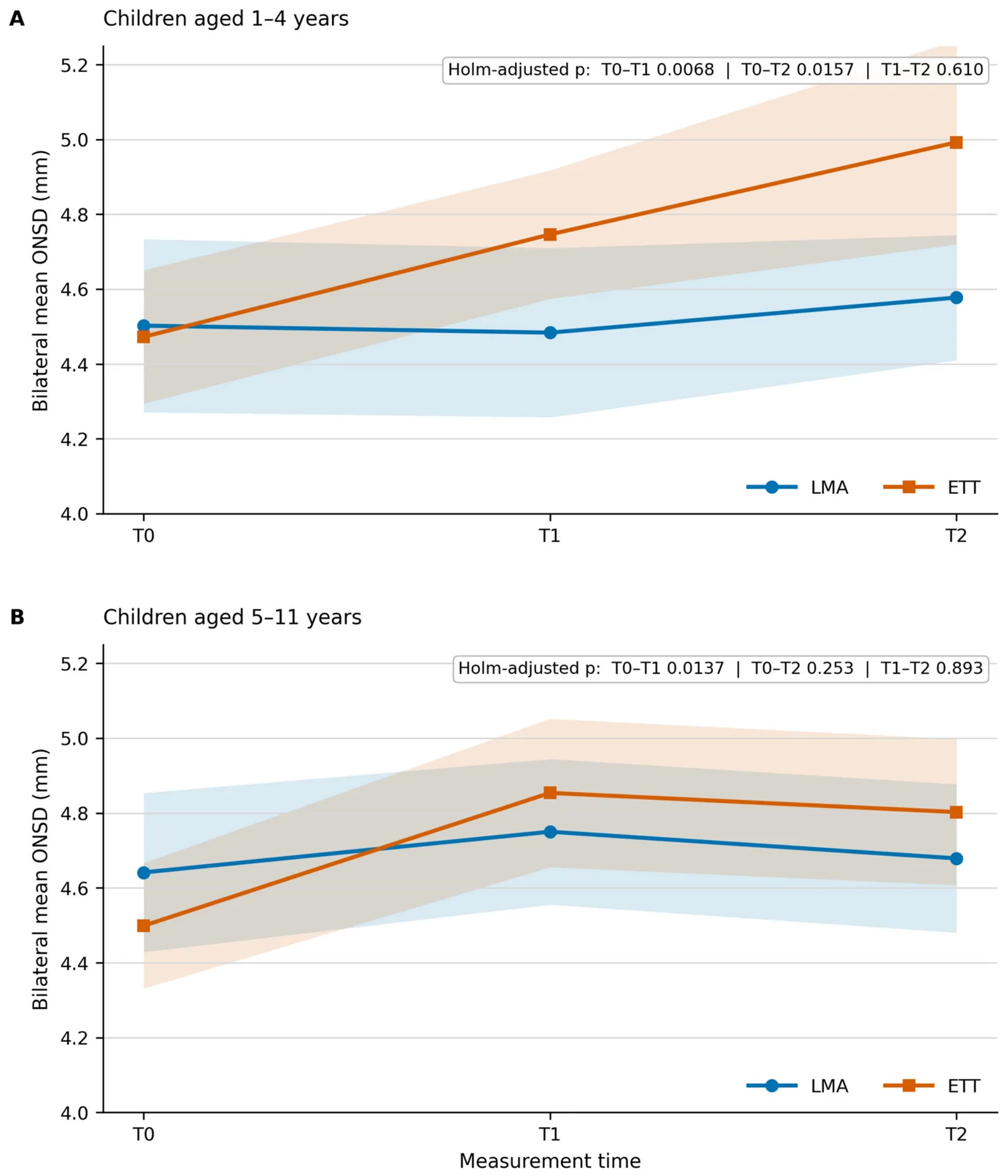
Time course of bilateral mean optic nerve sheath diameter according to airway device and age group. (**A**) Children aged 1–4 years; (**B**) children aged 5–11 years. Points show model-estimated marginal means, which were essentially identical to the observed group means (Table 3), and shaded bands show 95% confidence intervals. Insets report Holm-adjusted *p* values for between-device differences in change. T0, before airway placement; T1, after airway placement; T2, after airway removal; ETT, endotracheal tube; LMA, laryngeal mask airway; ONSD, optic nerve sheath diameter.

For left and right ONSD, the overall ETT–LMA differences in change from T0 to T1 were 0.276 mm (95% CI, 0.096–0.457; Holm-adjusted *p* = 0.0054) and 0.263 mm (95% CI, 0.076–0.449; Holm-adjusted *p* = 0.0115), respectively; tests of effect modification by age were not significant (Supplementary Table S1). The temporal course of ONSD according to age group and airway device is shown in Figure 2.

### 3.3. Intraoperative Variables

**Figure 3:**
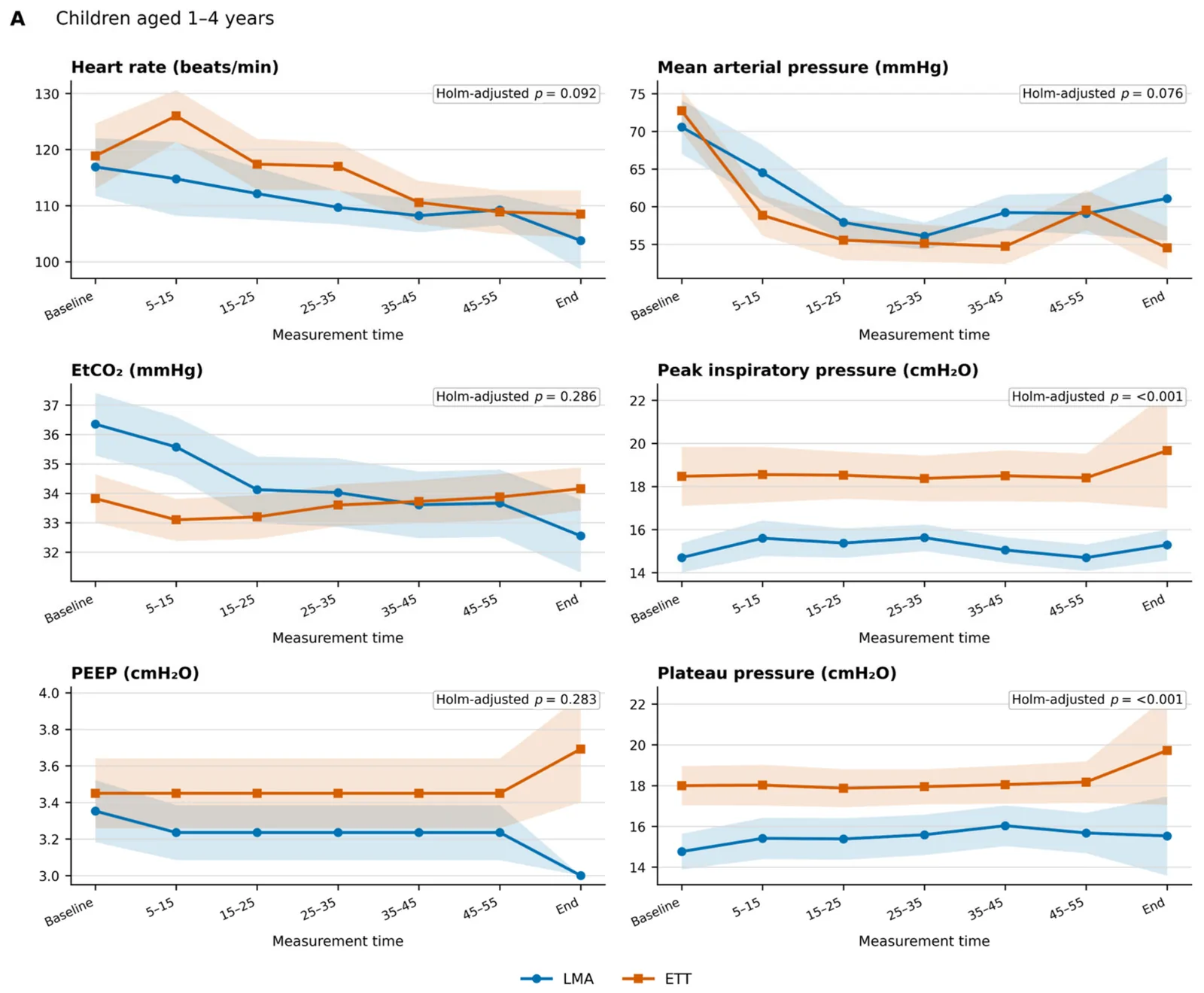

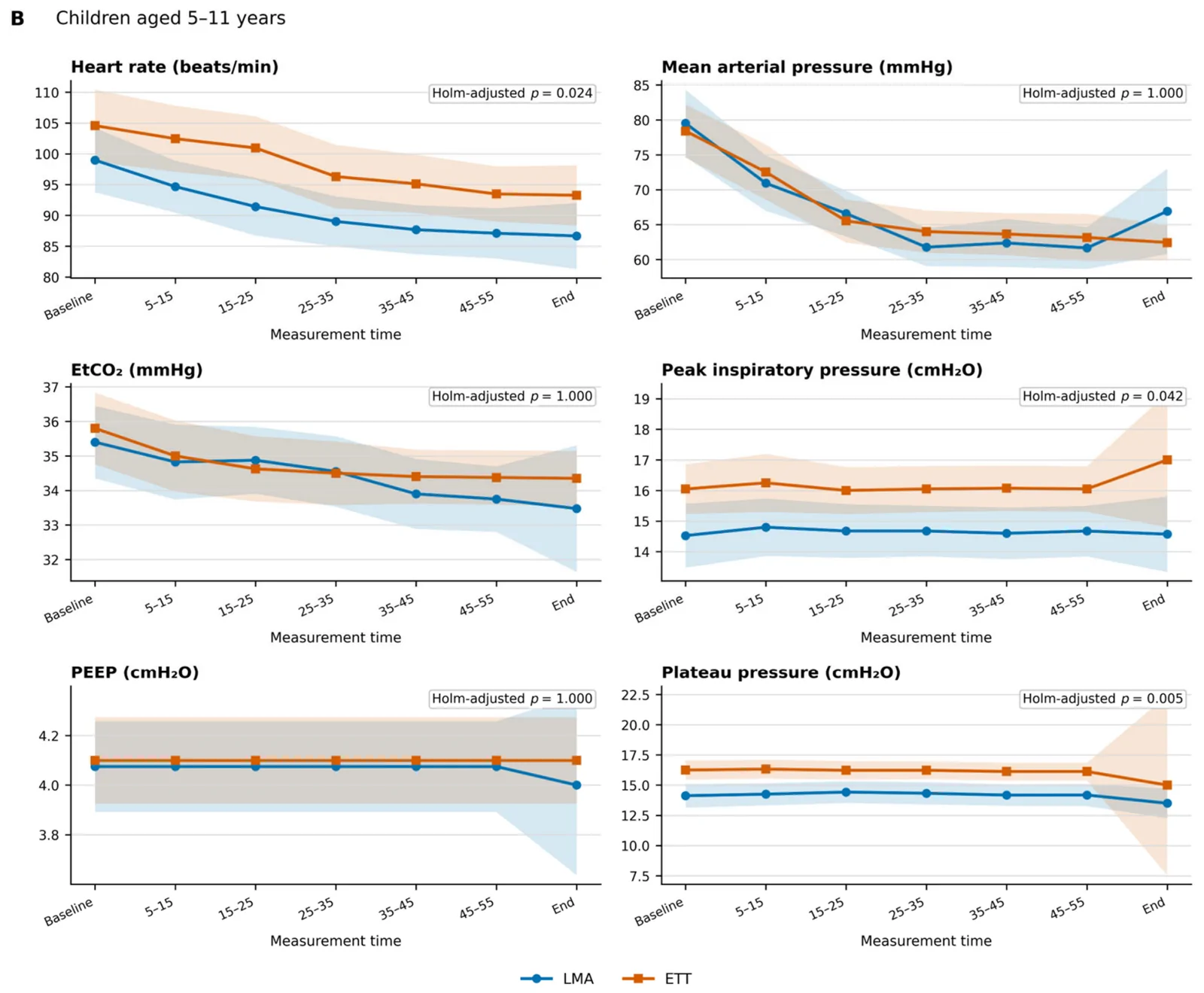
(**A**) Intraoperative physiological and ventilatory trends in children aged 1–4 years. Points represent arithmetic means, and shaded bands represent pointwise 95% confidence intervals. Holm-adjusted *p* values refer to time-averaged ETT–LMA contrasts; the adjustment included the seven predefined exploratory intraoperative outcomes. Sample sizes decreased at the end measurement. ETT, endotracheal tube; LMA, laryngeal mask airway; EtCO_2_, end-tidal carbon dioxide; PEEP, positive end-expiratory pressure. (**B**) Intraoperative physiological and ventilatory trends in children aged 5–11 years.

In the exploratory intraoperative analyses, seven outcomes were evaluated within the same analysis family using Holm adjustment. Time-averaged peak inspiratory pressure was higher in the ETT group than in the LMA group by 3.23 cmH_2_O in children aged 1–4 years (95% CI, 1.99–4.47; Holm-adjusted *p* < 0.001) and by 1.49 cmH_2_O in those aged 5–11 years (95% CI, 0.38–2.61; Holm-adjusted *p* = 0.042). The corresponding time-averaged differences in plateau pressure were 2.53 cmH_2_O (95% CI, 1.22–3.85; Holm-adjusted *p* < 0.001) and 1.98 cmH_2_O (95% CI, 0.84–3.11; Holm-adjusted *p* = 0.005). Heart rate was higher with ETT only in the 5–11-year age group (7.54 beats/min; 95% CI, 2.41–12.66; Holm-adjusted *p* = 0.024). Time-averaged differences in MAP, EtCO_2_, SpO_2_, and PEEP were not significant (Supplementary Table S2; Figure 3).

For EtCO_2_, the airway device × time interaction was nominally significant in the 1–4-year age group; however, the age group × airway device × time interaction did not remain significant after Holm adjustment (raw *p* = 0.032; Holm-adjusted *p* = 0.192). Therefore, time-specific EtCO_2_ comparisons were considered exploratory only. Because the number of observations at the end of anesthesia decreased, particularly for peak inspiratory and plateau pressures, estimates at this time point were interpreted cautiously.

## 4. Discussion

The principal finding of this prospective observational study was that the early increase in bilateral mean ONSD after airway placement was 0.269 mm greater in the ETT group than in the LMA group and remained statistically significant after Holm adjustment. The T0–T1 ETT–LMA differences in change were similar in direction and magnitude in the 1–4- and 5–11-year age groups. The nonsignificant three-way interaction, which directly compared the T0–T1 contrast between the age groups, indicates that there was no evidence that the early ONSD response differed by age group.

The ETT–LMA difference in change from T0 to T2 was statistically significant only in the 1–4-year age group. However, a significant result in one age group and a nonsignificant result in the other do not, by themselves, establish a difference in effect between age groups. Because no direct between-age contrast was performed to test whether the T0–T2 effect differed by age, this finding should not be interpreted as evidence of age-specific effect modification.

No significant ETT–LMA difference in change was identified from T1 to T2 in either age group. The similar direction and magnitude of the findings in the right- and left-eye analyses support the bilateral consistency of the primary result; however, measurement variability cannot be fully excluded because only one measurement was obtained from each eye.

In a study of pediatric ophthalmic surgery, ETT placement was associated with a greater increase in intraocular pressure than LMA placement [17]. Although this finding supports the possibility that tracheal intubation produces a stronger physiological stimulus, intraocular pressure is not equivalent to ONSD or intracranial pressure.

Yasmeen et al. reported a mean ONSD of 4.26 ± 0.44 mm after intubation in children aged 5–16 years [18]. In the same study of 23 children aged 5–16 years, Yasmeen et al. also showed that ONSD increased during laparoscopic surgery, consistent with evidence that ONSD can change during surgery itself. Because the T2 measurement encompassed the entire surgical course, which differed in case-mix between the groups, the T0–T2 comparison is particularly susceptible to time-dependent confounding and is therefore considered exploratory.

Pediatric studies evaluating elevated intracranial pressure have reported ONSD thresholds ranging from 4.91 to 5.75 mm across different age groups and measurement methods [19,20,21]. Because the present study was not a diagnostic-accuracy study and did not measure intracranial pressure, comparison with these thresholds is of limited value, and the group means cannot be interpreted as excluding clinical intracranial hypertension.

Adult studies evaluating intraocular pressure have reported greater pressure increases after ETT placement than after LMA placement [16,22]. Similarly, a systematic review and meta-analysis found that LMA use was associated with a lower intraocular pressure response than ETT use [23]. However, because these studies did not evaluate ONSD, they provide only indirect physiological context for the present findings.

In children aged 1–4 years, the increase in ONSD from baseline to T2 was 0.445 mm greater in the ETT group than in the LMA group. However, the absence of a significant between-group difference in the change from T1 to T2 does not support an additional increase in ONSD caused by airway removal in the ETT group. Because T2 was measured after surgery, surgical type and duration, ventilation, and emergence conditions may have influenced the result. Indeed, increases in ONSD have been reported during laparoscopic surgery and tonsillectomy involving use of a mouth gag [18,24]. Because surgical types were imbalanced between the airway groups, the T0–T2 finding should be considered exploratory.

As a hypothesis-generating observation, the higher peak inspiratory and plateau pressures observed in the ETT group in both age strata in the exploratory analyses may suggest a potential contribution of ventilatory mechanics.

Because the optic nerve sheath is continuous with the intracranial subarachnoid space, ONSD may reflect changes in intracranial pressure; however, it is not a direct pressure measurement [25]. Therefore, the between-group difference of 0.269 mm cannot be converted into a specific change in intracranial pressure.

The early increase in ONSD observed in the ETT group may reflect the combined effects of laryngoscopic and tracheal stimulation, airway pressures, and other perioperative factors. In a study in adults comparing tracheal intubation performed with different laryngoscopes, Küçükosman et al. found that airway instrumentation and laryngoscopy were associated with ONSD changes [14]. However, this evidence was obtained in adults and establishes plausibility rather than the mechanism underlying the present pediatric findings. Because the observational design did not permit separation of the independent effects of these components, the finding should be interpreted as an association between ETT use and change in ONSD. Differences in effect magnitude among studies may reflect differences in patient and surgical characteristics, measurement timing, ventilation strategy, and anesthetic protocol. Because airway pressures are post-exposure variables, any inference that they mediate the change in ONSD is hypothesis-generating only, and the primary model was not adjusted for them.

### Strengths and Limitations

The strengths of this study include balanced age-by-airway cells, bilateral repeated measurements, a clearly defined primary contrast, a mixed-effects model accounting for within-patient dependence, presentation of effect estimates with 95% confidence intervals, and adjustment for multiple comparisons.

This study has several limitations. The airway device was not randomized, creating the potential for selection bias, and surgical type and body weight were imbalanced across groups. Although the post hoc sensitivity analysis restricted to urological cases supported the primary finding, it cannot exclude unmeasured confounding. The investigator performing the measurements was not blinded to the airway device, which may have introduced measurement bias. Because measurements were not repeated independently, interobserver reliability could not be assessed; because only one measurement was obtained from each eye, within-examination repeatability could not be evaluated. T1 and T2 were not standardized using a timer, and differences in spontaneous or mask-assisted ventilation and level of arousal at T2 may have affected the measurements. Induction agents and ventilator settings were not fully standardized, which may also have resulted in residual confounding. Although the sample size calculation was based on an omnibus group × time interaction, the primary analysis tested a specific T0–T1 contrast. It is not known whether the observed 0.269 mm difference exceeds pediatric measurement error or a clinically meaningful change threshold. Because invasive intracranial pressure and neurological outcomes were not assessed, changes in ONSD cannot be interpreted as evidence of clinical intracranial hypertension. Finally, the single-center design and inclusion of only patients with ASA physical status I–II limit generalizability. Because airway selection was not randomized, confounding by indication is the principal limitation; surgical case-mix and body weight were imbalanced between groups, and all ear–nose–throat cases were in the ETT groups. Nevertheless, the primary association remained consistent in direction and significance in a sensitivity model adjusted for age, body weight, sex, and surgical category and in the urology-restricted subgroup; residual, unmeasured confounding cannot be excluded. Peak and plateau pressures were measured after device placement and are post-exposure (potential mediator) variables, for which the primary model was not adjusted. Excluding the LMA-to-ETT conversions and repeated-intubation cases may have removed patients with potentially larger physiological responses, biasing the primary effect toward the null. Because a single measurement was obtained per eye and images were not archived, measurement repeatability could not be quantified; it therefore remains unknown whether the 0.269 mm difference can be reliably distinguished from measurement error. Although the T1 measurement was obtained within approximately 1 min of airway placement, the exact interval was not recorded with a timer, and ETT cuff pressure was adjusted according to routine practice but not systematically recorded for analysis. Moreover, conversion from an LMA to an ETT is itself an outcome related to airway selection and clinical circumstances, so these post-selection exclusions are most likely to have attenuated rather than inflated the observed between-device difference. Because reliable, age-specific ONSD cut-off values are difficult to establish in children—owing to individual anatomical variability and interobserver measurement variability—ONSD may be better suited to intraoperative trend monitoring, based on the change from an individual baseline, than to threshold-based classification. Accordingly, future studies with standardized, blinded, and repeated measurements should evaluate whether serial ONSD can serve as an additional non-invasive component of anaesthetic monitoring in pediatric patients.

## 5. Conclusions

In the early period after airway placement, ETT use was associated with a greater increase in bilateral mean ONSD than LMA use. Although this association was directionally consistent in both age strata, there was no evidence that its magnitude differed by age group. Serial ultrasonographic ONSD measurements differed between children managed with an ETT and those managed with an i-gel in the early period after airway placement; this association was attenuated but persisted after adjustment for age, body weight, sex, and surgical category. The clinical significance of this small difference and its relationship with intracranial pressure remain uncertain.

## Figures and Tables

**Table 1:** Demographic and baseline characteristics.

Variable	1–4 Years/LMA (n = 40)	1–4 Years/ETT (n = 40)	5–11 Years/LMA (n = 40)	5–11 Years/ETT (n = 40)
Age, years	2.38 ± 0.87	2.17 ± 1.08	7.75 ± 1.93	7.08 ± 1.67
Body weight, kg	15.07 ± 3.20	13.20 ± 2.91	29.45 ± 10.40	26.30 ± 9.87
Female, n (%)	11 (27.5)	9 (22.5)	10 (25.0)	8 (20.0)
T0 bilateral mean ONSD, mm	4.50 ± 0.72	4.47 ± 0.56	4.64 ± 0.66	4.50 ± 0.52
Initial heart rate, beats/min	116.90 ± 16.08	118.88 ± 18.07	98.97 ± 16.28	104.58 ± 18.28
Initial MAP, mmHg	70.57 ± 11.14	72.74 ± 8.66	79.52 ± 15.07	78.41 ± 11.95
Initial EtCO_2_, mmHg	36.35 ± 3.32	33.83 ± 2.56	35.40 ± 3.28	35.80 ± 3.24
Initial peak inspiratory pressure, cmH_2_O	14.70 ± 2.14	18.48 ± 4.28	14.53 ± 3.27	16.05 ± 2.53
Initial PEEP, cmH_2_O	3.35 ± 0.49 ^1^	3.45 ± 0.60	4.08 ± 0.57	4.10 ± 0.55
Initial plateau pressure, cmH_2_O	14.76 ± 2.50 ^1^	18.00 ± 3.00	14.12 ± 3.06	16.25 ± 2.47

Data are mean ± standard deviation or n (%). ^1^ Initial PEEP and initial plateau pressure were not recorded in all patients of the 1–4 years/LMA group; these two values are based on 34 of the 40 patients (n = 34), as the earliest intraoperative ventilatory measurements were missing in 6 patients. All other values in this column are based on the full group (n = 40). ETT, endotracheal tube; LMA, laryngeal mask airway; ONSD, optic nerve sheath diameter; MAP, mean arterial pressure; EtCO_2_, end-tidal carbon dioxide; PEEP, positive end-expiratory pressure.

**Table 2:** Distribution of surgical types across study groups.

Group	Urology	ENT	Orthopedic/Plastic	General Surgery	Total
1–4 years/LMA	40 (100.0)	0	0	0	40
1–4 years/ETT	28 (70.0)	9 (22.5)	2 (5.0)	1 (2.5)	40
5–11 years/LMA	36 (90.0)	0	3 (7.5)	1 (2.5)	40
5–11 years/ETT	19 (47.5)	14 (35.0)	5 (12.5)	2 (5.0)	40
Total	123 (76.9)	23 (14.4)	10 (6.3)	4 (2.5)	160

Data are n (%); percentages are within study groups. The distribution was compared using Pearson’s chi-square test with a Monte Carlo *p* value because of sparse cells (χ^2^ = 40.79; df = 9; *p* < 0.001). ENT, ear, nose, and throat surgery; ETT, endotracheal tube; LMA, laryngeal mask airway.

**Table 3:** Model-based marginal means of bilateral mean ONSD over time.

Age Group	Airway	T0, mm	T1, mm	T2, mm	T1–T0 Change, mm	T2–T0 Change, mm
1–4 years	LMA	4.5	4.48	4.58	−0.019	0.075
1–4 years	ETT	4.47	4.75	4.99	0.274	0.52
5–11 years	LMA	4.64	4.75	4.68	0.109	0.038
5–11 years	ETT	4.5	4.85	4.8	0.355	0.304

T0, before airway placement; T1, after airway placement; T2, after airway removal; ETT, endotracheal tube; LMA, laryngeal mask airway; ONSD, optic nerve sheath diameter.

**Table 4:** Model-based contrasts in bilateral mean ONSD.

Contrast	Estimate, mm	95% CI, mm	Raw *p* Value	Holm-Adjusted *p* Value
Primary: overall ETT–LMA difference in T1–T0 change	0.269	0.131–0.408	<0.001	<0.001
Three-way interaction: difference in the T1–T0 ETT–LMA contrast between the 5–11- and 1–4-year age groups	−0.046	−0.323–0.231	0.743	0.743
ETT–LMA difference in T1–T0 change: 1–4 years	0.293	0.097–0.488	0.0034	0.0068
ETT–LMA difference in T1–T0 change: 5–11 years	0.246	0.050–0.442	0.0137	0.0137
ETT–LMA difference in T2–T0 change: 1–4 years	0.445	0.143–0.747	0.0039	0.0157
ETT–LMA difference in T2–T0 change: 5–11 years	0.266	−0.036–0.569	0.0843	0.2530
ETT–LMA difference in T2–T1 change: 1–4 years	0.153	−0.139–0.444	0.3050	0.6100
ETT–LMA difference in T2–T1 change: 5–11 years	0.020	−0.271–0.311	0.8930	0.8930

Positive ETT–LMA values indicate a greater increase in the ETT group. CI, confidence interval; ETT, endotracheal tube; LMA, laryngeal mask airway; ONSD, optic nerve sheath diameter. Holm-adjusted *p* values were calculated within predefined analysis families adopted for the final analysis.

## Data Availability

The data presented in this study are available from the corresponding author upon reasonable request. The data are not publicly available because of institutional data-protection and ethics-committee restrictions on sharing individual patient data; de-identified data and analysis code are available from the corresponding author upon reasonable request.

## Supplementary Materials

The following supporting information can be downloaded at: https://www.mdpi.com/article/10.3390/jcm15218345/s1, Table S1: Supportive analyses of left and right ONSD; Table S2: Time-averaged intraoperative ETT–LMA contrasts; Table S3: Predefined comparison families adopted for the final analysis and used for Holm adjustment.
