# Supplementary material for: Early Changes in Optic Nerve Sheath Diameter Associated with Endotracheal Tube Versus Laryngeal Mask Airway in Children Undergoing Elective Surgery Under General Anesthesia: A Prospective Observational Study
Source: J Clin Med. 2026 Oct 27;15(21):8345. doi: 10.3390/jcm15218345 (PMC13763705; doi:10.3390/jcm15218345)
Supplement: Supplementary file 1 [file jcm-15-08345-s001.zip › jcm-4600067-supplementary.pdf]

Supplementary Materials

Early Changes in Optic Nerve Sheath Diameter Associated with Endotracheal Tube versus Laryngeal Mask Airway in Children Undergoing Elective Surgery Under General Anesthesia: A Prospective Observational Study

Manuscript ID: jcm-4600067

Supplementary Table S1. Supportive analyses of left and right ONSD

Outcome	Overall T1–T0 ETT–LMA difference, mm	95% CI	Raw p	Holm-adjusted p	Holm p for age interaction
Left ONSD	0.276	0.096–0.457	0.003	0.0054	0.654
Right ONSD	0.263	0.076–0.449	0.006	0.0115	0.357

Positive ETT–LMA values indicate a greater increase in the ETT group. CI, confidence interval; ETT, endotracheal tube; LMA, laryngeal mask airway; ONSD, optic nerve sheath diameter.

Supplementary Table S2. Time-averaged intraoperative ETT–LMA contrasts

Outcome	1–4 years ETT–LMA difference (95% CI)	Holm p	5–11 years ETT–LMA difference (95% CI)	Holm p
Heart rate, beats/min	4.97 (0.68–9.27)	0.092	7.54 (2.41–12.66)	0.024
MAP, mmHg	−2.55 (−4.62 to −0.49)	0.076	0.24 (−3.01–3.50)	1.000
$EtCO_2$, mmHg	−0.81 (−1.90–0.27)	0.286	0.31 (−0.88–1.49)	1.000
SpO_2, percentage points	0.04 (−0.01–0.08)	0.286	0.00 (0.00–0.00)	1.000
Peak inspiratory pressure, cmH_2O	3.23 (1.99–4.47)	<0.001	1.49 (0.38–2.61)	0.042
PEEP, cmH_2O	0.20 (−0.03–0.43)	0.283	0.03 (−0.22–0.27)	1.000
Plateau pressure, cmH_2O	2.53 (1.22–3.85)	<0.001	1.98 (0.84–3.11)	0.005

Values are time-averaged ETT–LMA differences (95% CI). Positive values indicate higher values in the ETT group. Holm p values were adjusted across seven exploratory intraoperative outcomes. MAP, mean arterial pressure; $EtCO_2$, end-tidal carbon dioxide; PEEP, positive end-expiratory pressure; ETT, endotracheal tube; LMA, laryngeal mask airway; CI, confidence interval.

Supplementary Table S3. Predefined comparison families adopted for the final analysis and used for Holm adjustment

Analysis family	Outcome	Comparisons included in the Holm family	No. of tests	Scope
Confirmatory primary analysis	Bilateral mean ONSD	Overall ETT–LMA difference in T1–T0 change; age group × airway device × time interaction contrast for T1–T0	2	Adjusted together
Predefined age-stratified primary analysis	Bilateral mean ONSD	ETT–LMA difference in T1–T0 change in children aged 1–4 years; corresponding difference in children aged 5–11 years	2	Adjusted together
Secondary ONSD analysis	Bilateral mean ONSD	ETT–LMA differences in T2–T0 and T2–T1 changes, evaluated separately in each of the two age groups	4	Adjusted together
Supporting left-eye analysis	Left ONSD	Overall ETT–LMA difference in T1–T0 change; age group × airway device × time interaction contrast for T1–T0	2	Adjusted within the left-eye analysis
Supporting right-eye analysis	Right ONSD	Overall ETT–LMA difference in T1–T0 change; age group × airway device × time interaction contrast for T1–T0	2	Adjusted within the right-eye analysis
Exploratory intraoperative analysis, 1–4 years	Seven physiological and ventilatory outcomes	Time-averaged ETT–LMA contrasts for heart rate, MAP, $EtCO_2$, SpO_2, peak inspiratory pressure, PEEP, and plateau pressure	7	Adjusted across outcomes within the 1–4-year age group
Exploratory intraoperative analysis, 5–11 years	Seven physiological and ventilatory outcomes	Time-averaged ETT–LMA contrasts for heart rate, MAP, $EtCO_2$, SpO_2, peak inspiratory pressure, PEEP, and plateau pressure	7	Adjusted across outcomes within the 5–11-year age group

Holm adjustment was applied separately within each predefined family; p values were not adjusted across different families. The intraoperative families comprised time-averaged device contrasts and did not include pointwise comparisons at individual measurement times. CI, confidence interval; ETT, endotracheal tube; LMA, laryngeal mask airway; MAP, mean arterial pressure; $EtCO_2$, end-tidal carbon dioxide; ONSD, optic nerve sheath diameter; PEEP, positive end-expiratory pressure; SpO_2, peripheral oxygen saturation.
